# Subjective memory complaints, vascular risk factors and psychological distress in the middle-aged: a cross-sectional study

**DOI:** 10.1186/1471-244X-11-108

**Published:** 2011-07-01

**Authors:** Matt B Paradise, Nick S Glozier, Sharon L Naismith, Tracey A Davenport, Ian B Hickie

**Affiliations:** 1 Brain & Mind Research Institute, The University of Sydney, Building F, 94 Mallet Street, Camperdown, NSW 2050, Australia; 2 Academic Research & Statistical Consulting, 5 Herbert Street, West Ryde, NSW 2114, Australia

## Abstract

**Background:**

Subjective memory complaints (SMC) are common but their significance is still unclear. It has been suggested they are a precursor of mild cognitive impairment (MCI) or dementia and an early indicator of cognitive decline. Vascular risk factors have an important role in the development of dementia and possibly MCI. We therefore aimed to test the hypothesis that vascular risk factors were associated with SMC, independent of psychological distress, in a middle-aged community-dwelling population.

**Methods:**

A cross-sectional analysis of baseline data from the 45 and Up Study was performed. This is a cohort study of people living in New South Wales (Australia), and we explored the sample of 45, 532 participants aged between 45 and 64 years. SMC were defined as 'fair' or 'poor' on a self-reported five-point Likert scale of memory function. Vascular risk factors of obesity, diabetes, hypertension, hypercholesterolemia and smoking were identified by self-report. Psychological distress was measured by the Kessler Psychological Distress Scale. We tested the model generated from a randomly selected exploratory sample (n = 22, 766) with a confirmatory sample of equal size.

**Results:**

5, 479/45, 532 (12%) of respondents reported SMC. Using multivariate logistic regression, only two vascular risk factors: smoking (OR 1.18; 95% CI = 1.03 - 1.35) and hypercholesterolaemia (OR 1.19; 95% CI = 1.04 - 1.36) showed a small independent association with SMC. In contrast psychological distress was strongly associated with SMC. Those with the highest levels of psychological distress were 7.00 (95% CI = 5.41 - 9.07) times more likely to have SMC than the non-distressed. The confirmatory sample also demonstrated the strong association of SMC with psychological distress rather than vascular risk factors.

**Conclusions:**

In a large sample of middle-aged people without any history of major affective illness or stroke, psychological distress was strongly, and vascular risk factors only weakly, associated with SMC, although we cannot discount psychological distress acting as a mediator in any association between vascular risk factors and SMC. Given this, clinicians should be vigilant regarding the presence of an affective illness when assessing middle-aged patients presenting with memory problems.

## Background

Subjective memory complaints (SMC) are common and strongly associated with age. Estimates of their community prevalence have ranged from 11% [1] in 65 to 85 year olds to over 88% in those over the age of 85 years [2]. In Australia, Jorm et al. [3] found a prevalence of 10% in those with an average age of 62 years. There is uncertainty regarding the significance of SMC. They may be an early marker of cognitive decline with an underlying pathological basis, a feature of normal ageing and/or a reflection of psychological distress.

Cross-sectional studies have not consistently found an independent association between SMC and objective cognitive impairment [4]. In contrast, longitudinal studies have reported a strong association between SMC and the subsequent development of dementia or cognitive decline over periods of one to seven years [4-7]. Support for the pathological basis of SMC is further supported by recent neuroimaging studies, which have reported that euthymic individuals with memory complaints, free from significant objective deficits have early signs of Alzheimer's Disease (AD) pathology on MRI, such as medial-temporal lobe atrophy [8,9].

Vascular risk factors such as diabetes, smoking, obesity, hypertension and hypercholesterolaemia are well established as risk factors in the development of dementia [10,11] and MCI [12-15]. The exact mechanism for this is unclear, but there is considerable interest in the vascular hypothesis of AD, where vascular risk factors lead to cerebral hypoperfusion and later neurodegeneration [16]. To our knowledge only two studies, both cross-sectional, have examined the relationship between vascular risk factors and SMC, with conflicting results [3,17]. Neither of these studies examined the SMC-vascular risk factor association as their primary analysis.

There is also a strong association between SMC and depression [18-20], such that several studies have reported that after adjustment for mood, there is no longer an association of SMC with objective memory deficits [3,20,21]. There is also an association between vascular risk factors and depression [22,23] and indeed cerebrovascular disease in depression is predictive of poor prognosis and progression to dementia [24]. Any observed relationship between vascular risk factors and SMC may therefore be confounded/mediated by depression. Identification of the relative contribution of vascular risk factors and depression as potentially modifiable determinants of SMC in older people may enable early intervention strategies to prevent subsequent cognitive decline [25] and dementia, guiding both primary and secondary prevention approaches [25].

The objective of this study is to examine the associations between SMC, vascular risk factors and psychological distress. Our hypothesis is that vascular risk factors will be associated with SMC. Further, that this association will be independent of psychological distress.

## Methods

We used data from the 45 and Up Study [26], a very large study of healthy ageing, in the state of New South Wales (NSW), Australia. As detailed elsewhere [26], participants were recruited through the Medicare Australia enrolment database, which provides almost complete coverage of the general population. Eligible individuals were mailed an invitation to take part, an information leaflet, the study questionnaire, a consent form and a reply paid envelope. The participation rate of the 45 and Up Study was approximately 18% for the first 100, 000 participants [26]. We gained permission from The Sax Institute to use data from the 45 and Up Study dataset and ethical approval had been granted from the relevant ethics committees.

We limited our cohort to those aged between 45 and 64 years to reflect the early intervention approach [25]. Additionally, by limiting the sample to this age-range we attempted to minimise the chances that the sample would include people with pre-existing dementia. For the initial analysis, we also excluded those who had reported having been diagnosed with a stroke or receiving psychiatric medication for depression or anxiety, because of the known cognitive sequelae of these conditions [27].

### The 45 and Up Study questionnaire

All measures were extracted from the 45 and Up Study questionnaire [see 28]. This contained questions about demographic information, vascular risk factors and psychological distress.

Age was grouped into five-year intervals (i.e. 45-49 years, 50-54 years, 55-59 years, 60-64 years) and education was grouped into three levels (low, medium, high), according to both a priori assumptions and observations of their odds ratios. The three education levels were determined by whether the individual had left school early without a leaving certificate, had completed high school or had gone on to attain tertiary qualifications.

Five vascular risk factors were able to be considered; the presence of obesity, diabetes, whether the person was a current smoker and whether the individual was currently being treated for hypertension or hypercholesterolaemia. Obesity was defined as a Body Mass Index (BMI) greater than or equal to 30, according to World Health Organisation standards. The BMI was imputed using the weight and height recorded [29]. The presence of diabetes was determined by the question; "*Has a doctor EVER told you that you have... diabetes?"*. The participant's smoking status was determined by the question *"Are you are regular smoker now?" *Treatment for hypertension and hypercholesterolaemia were determined by the questions "*In the last month have you been treated for high blood pressure?" *and "...*high blood cholesterol?*"

Psychological distress was assessed by the 10-item Kessler Psychological Distress Scale (K10) [30], which provides a global measure of distress based on depressive and anxiety symptoms experienced in the last four weeks. The cut-off scores were based on the 'Clinical Research Unit for Anxiety and Depression' levels [31] and have been validated by the Australian Bureau of Statistics [32,33]. Each item was scored from 1 for *'none of the time' *to 5 for *'all of the time'*. Scores for the ten items were then summed, yielding a minimum possible score of 10 and a maximum possible score of 50. Low scores of 10-15 indicate low levels of psychological distress, scores ranging from 16-29 'moderate' levels of psychological distress and high scores of 30-50 indicate 'severe' levels of psychological distress.

The primary outcome variable of SMC was identified using a five-point Likert scale in which participants were asked *"In general, how would you rate your memory?"*, with a choice of the following responses '1 - poor', '2 - fair', '3 - good', '4 - very good' or '5 - excellent'. Those rating their memory as 'fair' or 'poor' were defined as experiencing SMC. This approach and cut-off point is consistent with previous studies that have examined SMC [34,35].

### Statistical analyses

All data were analysed using the Statistical Package for the Social Sciences (SPSS 17.0 for Windows, Chicago, USA). Only those participants with full data were included in the statistical analyses.

We generated baseline characteristics of the 45, 532 participants. Two univariate analyses were run. Firstly, we examined the associations between demographic information, vascular risk factors and psychological distress with SMC. We then analysed the association between vascular risk factors and psychological distress. For dichotomous and categorical variables, odds-ratios of their association with SMC were produced. High education was used as the reference group based on the a priori assumption that lower education would be associated with SMC.

The SPSS Random Number Generator was used to create two separate datasets of equal size (n = 22, 766) for exploratory and confirmatory model analyses. Chi-squared tests were used to determine if there was any significant difference in demographic information, vascular risk factors or psychological distress between the exploratory and confirmatory samples.

All variables were then entered into exploratory logistic regression analyses, using the 'enter' method. There were four models generated. Model 1 considered demographic variables. Model 2 used demographic variables and the measure of psychological distress. Model 3 included demographic variables and vascular risk factors. The final model, Model 4, included demographic variables, vascular risk factors and a measure of psychological distress.

Finally, based on sound statistical results and *a priori *hypotheses, Model 4 was considered to be the most robust and was subsequently imposed on the confirmatory dataset to test its validity. For all analyses, we took the conservative approach of setting the significance level at p < 0.001, to reduce the chance of a Type-1 error given our large sample size.

## Results

Of the 103, 041 total respondents, 55, 685 were aged less than 65 years and had not had a stroke or received treatment for depression and anxiety within the last month. Of these, 45, 533 participants had complete data available for all variables.

Demographic, vascular risk factor and psychological distress characteristics are shown in Table 1. SMC were strongly associated with low education and male gender, the presence of diabetes, being a current smoker and receiving treatment for hypercholesterolaemia. Obesity and receiving treatment of hypertension were not associated with SMC. Psychological distress had the strongest association with SMC. Those with the greatest psychological distress (i.e. K10 category of 'severe') had an odds ratio of 7.68 (95% CI = 6.38 - 9.24) of having SMC compared to those with the least psychological distress.

**Table 1:** Characteristics of the 45 and Up Study sample and association of variables with SMC, N = 45, 532

	Total	SMC (n, column %)	No SMC (n, column %)	Odds ratio (95% CI)
				
**N**	**45, 532**	**5, 479 (12.0%)**	**40, 053 (88.0%)**	
**Demographics**				
Age				
-45 to 49 years	9, 582	1, 152 (21.0%)	8, 430 (21.0%)	1.00
-50 to 54 years	12, 237	1, 441 (26.3%)	10, 796 (27.0%)	0.98 (0.90 - 1.06)
-55 to 59 years	12, 712	1, 510 (27.6%)	11, 202 (28.0%)	0.99 (0.91 - 1.07)
-60 to 64 years	11, 001	1, 376 (25.1%)	9, 625 (24.0%)	1.05 (0.96 - 1.14)
Gender				
-Male	20, 606	2, 674 (48.8%)	17, 932 (44.8%)	1.18 (1.11 - 1.24)*
-Female	24, 926	2, 805 (51.2%)	22, 121 (55.2%)	
Education				
-High	13, 743	1, 071 (19.5%)	12, 672 (31.6%)	1.00
-Medium	19, 856	2, 462 (44.9%)	17, 394 (43.4%)	1.68 (1.55 - 1.81)*
-Low	11, 933	1, 946 (35.5%)	9, 987 (24.9%)	2.31 (2.13 - 2.50)*
**Vascular risk factors**				
Obesity	10, 147	1, 306 (23.8%)	8, 841 (22.1%)	1.11 (1.03 - 1.18)
-non-obesity	35, 385	4, 173 (76.2%)	31, 212 (77.9%)	
Diabetes	2, 475	379 (6.9%)	2, 096 (5.2%)	1.35 (1.20 - 1.51)*
-no diabetes	43, 057	5, 100 (93.1%)	37, 957 (94.8%)	
Current smoker	3, 928	607 (11.1%)	3, 321 (8.3%)	1.38 (1.26 - 1.51)*
-non-smoker	41, 604	4, 872 (88.9%)	36, 732 (91.7%)	
Treatment for hypertension	7, 338	928 (16.9%)	6, 410 (16.0%)	1.07 (0.99 - 1.15)
-no treatment for hypertension	38, 194	4, 551 (83.1%)	33, 643 (84.0%)	
Treatment for hypercholesterolaemia	4, 892	692 (12.6%)	4, 200 (10.5%)	1.23 (1.13 - 1.35)*
-no treatment for hypercholesterolaemia	40, 640	4, 787 (87.4%)	35, 853 (89.5%)	
**Psychological distress**				
K10-low level of distress	35, 713	3, 139 (57.3%)	32, 574 (81.3%)	1.00
- moderate level of distress	9, 344	2, 138 (39.0%)	7, 206 (18.0%)	3.08 (2.90 - 3.27)*
- severe level of distress	475	202 (3.7%)	273 (0.7%)	7.68 (6.38 - 9.24)*

Note: * p < 0.001.

Table 2 shows the association of vascular risk factors with psychological distress. Obesity, diabetes, being a current smoker and receiving treatment for hypercholesterolaemia were associated with psychological distress.

**Table 2:** Association of vascular risk factors with psychological distress, N = 45, 532

		Level of psychological distress - K10			
Vascular risk factors	N		n (%)		Chi-square
			
		Low	Moderate	Severe	
Obesity	10, 147	7, 629 (75.2%)	2, 363 (23.3%)	155 (1.5%)	95.61*
-non-obesity	35, 385	28, 084 (79.4%)	6, 981 (19.7%)	320 (0.9%)	
Diabetes	2, 475	1, 821 (73.6%)	599 (24.2%)	55 (2.2%)	60.03*
-no diabetes	43, 057	33, 892 (78.7%)	8, 745 (20.3%)	420 (1.0%)	
Current smoker	3, 928	2, 752 (70.1%)	1, 065 (27.1%)	111 (2.8%)	260.39*
-non-smoker	41, 604	32, 961 (79.2%)	8, 279 (19.9%)	364 (0.9%)	
Treatment for hypertension	7, 338	5, 653 (77.0%)	1, 607 (21.9%)	78 (1.1%)	10.30
-no treatment for hypertension	38, 194	30, 060 (78.7%)	7, 737 (20.3%)	397 (1.0%)	
Treatment for hypercholesterolaemia	4, 892	3, 731 (76.3%)	1, 098 (22.4%)	63 (1.3%)	16.30*
-no treatment for hypercholesterolaemia	40, 640	31, 892 (78.7%)	8, 246 (20.3%)	412 (1.0%)	

Note: *p < 0.001.

There were no significant differences in any of the baseline characteristics between the exploratory and confirmatory samples. Table 3 shows Models 1, 2, 3 and 4 of the multivariate analysis generated with the exploratory sample. Model 1 demonstrates that male gender and low education, but not age, were associated with SMC. These results were not attenuated by the presence of psychological distress in Model 2, which was strongly associated with SMC. Model 3 demonstrates that once adjusted for demographic variables, the only factor vascular risk factor that remained significantly associated with SMC in our conservative approach was being a current smoker (being treated for hypercholesterolaemia and diabetes both showed a trend towards association with SMC; p = 0.002). When adjusted for the presence of psychological distress in Model 4, the association between vascular risk factor and SMC further weakened, such that even in this very large sample, there were no statistically significant associations at the p < 0.001 level although both being a current smoker and hypercholesterolaemia treatment were associated at standard levels of significance. In all analyses, psychological distress had the strongest association with SMC. When fully adjusted, those with 'severe' psychological distress still had 7.00 times the odds (95% CI = 5.41 - 9.07) of SMC.

**Table 3:** Multivariate models of associations of SMC using the exploratory sample, N = 22, 766

	Model 1	Model 2	Model 3	Model 4
	Odds ratio (95% CI)	Odds ratio (95% CI)	Odds ratio (95% CI)	Odds ratio (95% CI)
**Demographics**				
Age				
-45 to 49 years	1.00	1.00	1.00	1.00
-50 to 54 years	0.97 (0.86 - 1.09)	1.01 (0.90 - 1.14)	0.97 (0.86 - 1.09)	1.01 (0.90 - 1.15)
-55 to 59 years	0.96 (0.85 - 1.08)	1.09 (0.96 - 1.22)	0.96 (0.85 - 1.08)	1.08 (0.96 - 1.22)
-60 to 64 years	0.95 (0.85 - 1.08)	1.09 (0.99 - 1.23)	0.95 (0.84 - 1.07)	1.08 (0.95 - 1.22)
Gender				
-Male	1.26 (1.16 - 1.36)*	1.29 (1.18 - 1.40)*	1.23 (1.13 - 1.34)*	1.27 (1.17 - 1.40)*
Education				
-High	1.00	1.00	1.00	1.00
-Medium	1.62 (1.46 - 1.80)*	1.59 (1.43 - 1.77)*	1.59 (1.43 - 1.77)*	1.58 (1.42 - 1.76)*
-Low	2.22 (1.98 - 2.48)*	2.06 (1.84 - 2.31)*	2.14 (1.91 - 2.40)*	2.03 (1.81 - 2.28)*
**Vascular risk factors**				
Obesity			0.98 (0.89 - 1.08)	0.94 (0.85 - 1.03)
Diabetes			1.21 (1.03 - 1.43)	1.13 (0.96 - 1.34)
Current smoker			1.31 (1.15 - 1.49)*	1.18 (1.03 - 1.35)
Treatment for hypertension			0.97 (0.86 - 1.09)	0.96 (0.85 - 1.08)
Treatment for hypercholesterolaemia			1.22 (1.07 - 1.39)	1.19 (1.04 - 1.36)
**Psychological distress**				
K10-low level of distress		1.00		1.00
- moderate level of distress		2.96 (2.71 - 3.23)*		2.94 (2.69 - 3.21)*
- severe level of distress		7.21 (5.57 - 9.32)*		7.00 (5.41 - 9.07)*

Notes: *p < 0.001; Model 1 - demographic variables; Model 2 - demographic variables and psychological distress; Model 3 - demographic variables and vascular risk factors; Model 4 - demographic variables, vascular risk factors and psychological distress.

The final Model 4 was imposed on into the confirmatory sample of 22, 766. This confirmed that male gender (OR 1.30; 95% CI = 1.19 - 1.41) and low education (OR 1.68; 95% CI = 1.51 - 1.88) were associated with SMC, but age was not. As seen in the exploratory dataset, in the presence of both demographic variables and psychological distress, no vascular risk factors were associated with SMC. Severe psychological distress was again strongly associated with SMC with a similar odds ratio of 6.86 (95% CI = 5.20 - 9.05).

## Discussion

In an Australian community sample of 45 to 64 year olds, who are not currently receiving treatment for depression or anxiety and who are unlikely to have significant cognitive impairment, SMC are common, with a prevalence of 12%. In univariate analysis, the vascular risk factors of diabetes, being a current smoker and treatment for hypercholesterolaemia were associated with SMC. In multivariate analyses, when adjusted for psychological distress and demographics, vascular risk factors showed only weak associations with SMC.. This may be because of the confounding effect of gender and education, with post-hoc analyses showing male gender and less education were strongly associated with the presence of vascular risk factors.

The lack of strong association of vascular risk factors with SMC is consistent with Jorm et al. [3], who reported that diabetes, 'heart troubles' and a history of strokes were not associated with memory complaints in multivariate analysis, in an Australian sample of community-dwelling 60 to 64 years old with generally good cognition. This is also consistent with Stewart et al. [17] who found that in an Afro-Caribbean population hypertension, diabetes, electrocardiography-defined ischemia, cholesterol or triglyceride levels were not associated with memory complaints (although having had a stroke was a significant risk).

In contrast to vascular risk factors, there was a strong independent association between psychological distress and SMC. This is consistent with other literature [18-20] and may reflect the common depressive symptoms of poor memory and concentration. There may also be a tendency in subjects with significant psychological distress to have a negative attribution bias and therefore over-report memory complaints [36]. Memory complaints in those with high levels of psychological distress may also represent a common underlying pathophysiology. Depression, for example is now recognised as an independent modifiable risk-factor for cognitive decline [37] and conversion of MCI to dementia [38]. Several mechanisms have been postulated for this relationship including the neurotoxic effects of chronic hypercortisolaemia, reduced levels of neurotrophic factors [39], alterations in glial-neuronal networks, vascular disease and inflammatory processes [40]. Indeed, older patients with depression have reduced hippocampal size, which in turn, is associated with poorer memory [40].

Our data shows a strong association between vascular risk and psychological distress. This is consistent with the literature, where the association between vascular risk factors and depression is well documented [23]. We hypothesised that psychological distress might mediate any relationship between vascular risk factors and SMC. However, the general lack of associations between vascular risk factors and SMC seen in Model 3 would suggest that any such mediation is minimal. Further exploration of these complex relationships is warranted in longitudinal studies.

There are several strengths of this study. It is the largest study to date that examines the relationship between psychological distress, vascular risk factors and SMC. The questions were all taken from validated questionnaires used extensively in Australian populations. Our finding of a SMC prevalence of 12% is consistent with the community samples from the literature [1,3].

The major limitation of this study is the uncertainty regarding the direction of causality of any observed association: for example, it may be that SMC lead to psychological distress, rather than the other way around. We also cannot correlate the measure of SMC with an objective cognitive assessment. We could not specifically exclude cases of dementia or MCI although by limiting the cohort to those aged less than 65, we are unlikely to have many cases. A recent meta-analysis found a dementia prevalence rate of 0.6% for those aged between 60 and 64 years in Australasia [41]. Also, the ability to complete, sign and return the questionnaire would exclude those with significant cognitive decline. In any event, as seen with stroke, these cases may be unlikely to dramatically affect the results.

The presence of hypertension and hypercholesterolaemia were determined by the prescription of medication for these conditions. There may therefore be undetected individuals with these vascular risk factors and those receiving treatments for these conditions may paradoxically be at a reduced risk.

Finally, there is the effect of the size of the study. Analyses of such large samples may result in Type I errors. Such studies always result in a trade-off between efficiency and the diminution of measurement errors in a large sample against the ability of such measures to provide valid evaluations at an individual level. Although we do not anticipate any significant bias in the response, the error will serve to reduce the observed estimate of the association. The similar results found in the exploratory and confirmatory datasets strengthen the validity of our conclusions.

All our measures are self-report and as such, our exposure may be subject to information bias, with those people reporting SMC potentially being less likely to recall the presence of vascular risk factors. This would lead us to have underestimated any real association between vascular risk factors and SMC.

The participation rate of the 45 and Up Study was low, at 18% for the first 100, 000 participants. Although this raises questions about the representativeness of the sample, comparison with the NSW Population Health Survey demonstrated good generalisability [42].

## Conclusions

SMC are common in community-dwelling middle-aged adults without any history of major affective illness or stroke. Vascular risk factors were not independently associated with SMC. Psychological distress was highly associated with SMC as well as with vascular risk factors.

This finding adds some support to the concept of vascular depression [43] and emphasises the need for clinicians to take SMC seriously in their patients, as a common indicator of undetected psychological distress and possible affective illness. This may best be achieved through primary care education programmes highlighting early detection and management of psychological distress in at-risk groups [44,45].

The complex relationship between memory complaints, vascular risk and psychological distress needs further exploration in longitudinal studies. A greater understanding of SMC may allow early intervention to prevent psychological distress and potentially modify cognitive decline.

## Competing interests

The authors declare that they have no competing interests.

## Authors' contributions

MBP conceived the study and wrote the first draft. NSG helped with the study design, statistics and editing the manuscript. SLN provided input into the study design and helped draft the manuscript. TAD provided statistical advice and helped edit the manuscript. IBH provided overall supervision for the project and helped draft the manuscript. All authors read and approved the final manuscript.

## Pre-publication history

The pre-publication history for this paper can be accessed here:

http://www.biomedcentral.com/1471-244X/11/108/prepub

## References

[B1] Geerlings MI Jonker C Bouter LM Ader HJ Schmand B Association between memory complaints and incident Alzheimer's disease in elderly people with normal baseline cognition Am J Geriatr Psychiatry 1999 156 4 531 537 10.1176/ajp.156.4.531 10200730

[B2] Larrabee GJ Crook TH Estimated prevalence of age-associated memory impairment derived from standardized tests of memory function Int Psychogeriatr 1994 6 1 95 104 10.1017/S1041610294001663 8054499

[B3] Jorm AF Butterworth P Anstey KJ Christensen H Easteal S Maller J Mather KA Turakulov RI Wen W Sachdev P Memory complaints in a community sample aged 60-64 years: associations with cognitive functioning, psychiatric symptoms, medical conditions, APOE genotype, hippocampus and amygdala volumes, and white-matter hyperintensities Psychol Med 2004 34 8 1495 1506 10.1017/S0033291704003162 15724880

[B4] Reid LM Maclullich AMJ Subjective memory complaints and cognitive impairment in older people Dement Geriatr Cogn Disord 2006 22 5-6 471 485 10.1159/000096295 17047326

[B5] Jessen F Wiese B Bachmann C Eifflaender-Gorfer S Haller F Kolsch H Luck T Mosch E van den Bussche H Wagner M Wollny A Zimmermann T Pentzek M Riedel-Heller SG Romberg H-P Weyerer S Kaduszkiewicz H Maier W Bickel H Prediction of dementia by subjective memory impairment: effects of severity and temporal association with cognitive impairment Arch Gen Psychiatry 67 4 414 422 10.1001/archgenpsychiatry.2010.30 20368517

[B6] Jonker C Geerlings MI Schmand B Are memory complaints predictive for dementia? A review of clinical and population-based studies Int J Geriatr Psychiatry 2000 15 11 983 991 10.1002/1099-1166(200011)15:11<983::AID-GPS238>3.0.CO;2-5 11113976

[B7] Reisberg B Prichep L Mosconi L John ER Glodzik-Sobanska L Boksay I Monteiro I Torossian C Vedvyas A Ashraf N Jamil IA de Leon MJ The pre-mild cognitive impairment, subjective cognitive impairment stage of Alzheimer's disease Alzheimers Dement 2008 4 1 Suppl 1 S98 S108 18632010 10.1016/j.jalz.2007.11.017

[B8] Saykin AJ Wishart HA Rabin LA Santulli RB Flashman LA West JD McHugh TL Mamourian AC Older adults with cognitive complaints show brain atrophy similar to that of amnestic MCI Neurology 2006 67 5 834 842 10.1212/01.wnl.0000234032.77541.a2 16966547 PMC3488276

[B9] Chao LL Mueller SG Buckley ST Peek K Raptentsetseng S Elman J Yaffe K Miller BL Kramer JH Madison C Mungas D Schuff N Weiner MW Evidence of neurodegeneration in brains of older adults who do not yet fulfill MCI criteria Neurobiol Aging 2008 31 3 368 377 18550226 10.1016/j.neurobiolaging.2008.05.004 PMC2814904

[B10] Luchsinger JA Reitz C Honig LS Tang MX Shea S Mayeux R Aggregation of vascular risk factors and risk of incident Alzheimer disease Neurology 2005 65 4 545 551 10.1212/01.wnl.0000172914.08967.dc 16116114 PMC1619350

[B11] Gorelick PB Risk factors for vascular dementia and Alzheimer disease Stroke 2004 35 11 Suppl 1 2620 2622 15375299 10.1161/01.STR.0000143318.70292.47

[B12] Kivipelto M Helkala EL Hanninen T Laakso MP Hallikainen M Alhainen K Soininen H Tuomilehto J Nissinen A Midlife vascular risk factors and late-life mild cognitive impairment: A population-based study Neurology 2001 56 12 1683 1689 11425934 10.1212/wnl.56.12.1683

[B13] Solfrizzi V Panza F Colacicco AM D'Introno A Capurso C Torres F Grigoletto F Maggi S Del Parigi A Reiman EM Caselli RJ Scafato E Farchi G Capurso A Vascular risk factors, incidence of MCI, and rates of progression to dementia Neurology 2004 63 10 1882 1891 15557506 10.1212/01.wnl.0000144281.38555.e3

[B14] Ravaglia G Forti P Maioli F Martelli M Servadei L Brunetti N Pantieri G Mariani E Conversion of mild cognitive impairment to dementia: predictive role of mild cognitive impairment subtypes and vascular risk factors Dement Geriatr Cogn Disord 2006 21 1 51 58 10.1159/000089515 16276110

[B15] Mariani E Monastero R Ercolani S Mangialasche F Caputo M Feliziani FT Vitale DF Senin U Mecocci P Re GSG Vascular risk factors in mild cognitive impairment subtypes. Findings from the ReGAl project Dement Geriatr Cogn Disord 2007 24 6 448 456 10.1159/000110653 17975314

[B16] de la Torre JC Is Alzheimer's disease a neurodegenerative or a vascular disorder? Data, dogma, and dialectics Lancet Neurol 2004 3 3 184 190 10.1016/S1474-4422(04)00683-0 14980533

[B17] Stewart R Russ C Richards M Brayne C Lovestone S Mann A Depression, APOE genotype and subjective memory impairment: a cross-sectional study in an African-Caribbean population Psychol Med 2001 31 3 431 440 11305851

[B18] Tobiansky R Blizard R Livingston G Mann A The Gospel Oak Study stage IV: the clinical relevance of subjective memory impairment in older people Psychol Med 1995 25 4 779 786 10.1017/S0033291700035029 7480455

[B19] Hanninen T Reinikainen KJ Helkala EL Koivisto K Mykkanen L Laakso M Pyorala K Riekkinen PJ Subjective memory complaints and personality traits in normal elderly subjects J Am Geriatr Soc 1994 42 1 1 4 8277103 10.1111/j.1532-5415.1994.tb06064.x

[B20] Minett TSC Dean JL Firbank M English P O'Brien JT Subjective memory complaints, white-matter lesions, depressive symptoms, and cognition in elderly patients Am J Geriatr Psychiatry 2005 13 8 665 671 16085782 10.1176/appi.ajgp.13.8.665

[B21] Minett TSC Da Silva RV Ortiz KZ Bertolucci PHF Subjective memory complaints in an elderly sample: a cross-sectional study Int J Geriatr Psychiatry 2008 23 1 49 54 10.1002/gps.1836 17520662

[B22] Hickie I Scott E Mitchell P Wilhelm K Austin MP Bennett B Subcortical hyperintensities on magnetic resonance imaging: clinical correlates and prognostic significance in patients with severe depression Biol Psychiatry 1995 37 3 151 160 10.1016/0006-3223(94)00174-2 7727623

[B23] Hickie I Scott E Late-onset depressive disorders: a preventable variant of cerebrovascular disease? Psychol Med 1998 28 5 1007 1013 10.1017/S0033291797006090 9794008

[B24] Hickie I Scott E Wilhelm K Brodaty H Subcortical hyperintensities on magnetic resonance imaging in patients with severe depression--a longitudinal evaluation Biol Psychiatry 1997 42 5 367 374 10.1016/S0006-3223(96)00363-0 9276077

[B25] Naismith SL Glozier NS Burke D Carter PE Scott E Hickie IB Early intervention for cognitive decline: is there a role for multiple medical or behavioural interventions? Early Interv Psychiatry 2009 3 19 27 10.1111/j.1751-7893.2008.00102.x 21352171

[B26] Banks E Redman S Jorm L Armstrong B Bauman A Beard J Beral V Byles J Corbett S Cumming R Harris M Sitas F Smith W Taylor L Wutzke S Lujic S Cohort profile: the 45 and up study Int J Epidemiol 2008 37 5 941 947 17881411 10.1093/ije/dym184 PMC2557061

[B27] Naismith SL Hickie IB Turner K Little CL Winter V Ward PB Wilhelm K Mitchell P Parker G Neuropsychological performance in patients with depression is associated with clinical, etiological and genetic risk factors J Clin Exp Neuropsychol 2003 25 6 866 877 10.1076/jcen.25.6.866.16472 13680463

[B28] The 45 and Up Study http://www.45andup.org.au

[B29] WHO Technical report series 894: Obesity: Preventing and managing the global epidemic 2000 Geneva: World Health Organisation 11234459

[B30] Kessler RC Andrews G Colpe LJ Hiripi E Mroczek DK Normand SLT Walters EE Zaslavsky AM Short screening scales to monitor population prevalences and trends in non-specific psychological distress Psychol Med 2002 32 6 959 976 10.1017/S0033291702006074 12214795

[B31] Clinical Research Unit for Anxiety and Depression (CRUfAD) http://www.crufad.org

[B32] 4817.0.55.001 - Information Paper: Use of the Kessler Psychological Distress Scale in ABS Health Surveys, Australia, 2001 http://www.abs.gov.au/ausstats/abs@.nsf/mf/4817.0.55.001#3.%20Scoring%20the%20K10

[B33] Andrews G Slade T Interpreting scores on the Kessler Psychological Distress Scale (K10) Aust N Z J Public Health 2001 25 6 494 497 10.1111/j.1467-842X.2001.tb00310.x 11824981

[B34] Purser JL Fillenbaum GG Wallace RB Memory complaint is not necessary for diagnosis of mild cognitive impairment and does not predict 10-year trajectories of functional disability, word recall, or short portable mental status questionnaire limitations J Am Geriatr Soc 2006 54 2 335 338 10.1111/j.1532-5415.2005.00589.x 16460388

[B35] Ganguli M Dodge HH Shen C DeKosky ST Mild cognitive impairment, amnestic type: an epidemiologic study Neurology 2004 63 1 115 121 15249620 10.1212/01.wnl.0000132523.27540.81

[B36] Naismith SL Longley WA Scott EM Hickie IB Disability in major depression related to self-rated and objectively-measured cognitive deficits: a preliminary study BMC Psychiatry 2007 7 32 10.1186/1471-244X-7-32 17634111 PMC1959228

[B37] Ownby RL Crocco E Acevedo A John V Loewenstein D Depression and risk for Alzheimer disease - Systematic review, meta-analysis, and metaregression analysis Arch Gen Psychiatry 2006 63 5 530 538 10.1001/archpsyc.63.5.530 16651510 PMC3530614

[B38] Modrego PJ Ferrandez J Depression in patients with mild cognitive impairment increases the risk of developing dementia of Alzheimer type: a prospective cohort study Arch Neurol 2004 61 8 1290 1293 10.1001/archneur.61.8.1290 15313849

[B39] Duman RS Monteggia LM A neurotrophic model for stress-related mood disorders Biol Psychiatry 2006 59 12 1116 1127 10.1016/j.biopsych.2006.02.013 16631126

[B40] Hickie I Naismith S Ward PB Turner K Scott E Mitchell P Wilhelm K Parker G Reduced hippocampal volumes and memory loss in patients with early- and late-onset depression Br J Psychiatry 2005 186 197 202 10.1192/bjp.186.3.197 15738499

[B41] Ferri CP Prince M Brayne C Brodaty H Fratiglioni L Ganguli M Hall K Hasegawa K Hendrie H Huang YQ Jorm A Mathers C Menezes PR Rimmer E Scazufca M Global prevalence of dementia: a Delphi consensus study Lancet 2005 366 9503 2112 2117 16360788 10.1016/S0140-6736(05)67889-0 PMC2850264

[B42] Mealing NM Banks E Jorm LR Steel DG Clements MS Rogers KD Investigation of relative risk estimates from studies of the same population with contrasting response rates and designs BMC Med Res Methodol 10 26 10.1186/1471-2288-10-26 PMC2868856 20356408

[B43] Alexopoulos GS Meyers BS Young RC Campbell S Silbersweig D Charlson M 'Vascular depression' hypothesis Arch Gen Psychiatry 1997 54 10 915 922 9337771 10.1001/archpsyc.1997.01830220033006

[B44] Hickie IB Davenport TA Scott EM Hadzi-Pavlovic D Naismith SL Koschera A Unmet need for recognition of common mental disorders in Australian general practice Med J Aust 2001 175 24 16 10.5694/j.1326-5377.2001.tb143785.x 11556431

[B45] Naismith SL Hickie IB Scott EM Davenport TA Effects of mental health training and clinical audit on general practitioners' management of common mental disorders Med J Aust 2001 175 7 16 10.5694/j.1326-5377.2001.tb143789.x 11556436

